# Application Value of a Novel Micro-Coil in High-Resolution Imaging of Experimental Mice Based on 3.0 T Clinical MR

**DOI:** 10.3390/tomography10060064

**Published:** 2024-06-01

**Authors:** Xueke Qiu, Yang Liu, Fajin Lv

**Affiliations:** 1 State Key Laboratory of Ultrasound in Medicine and Engineering, College of Biomedical Engineering, Chongqing Medical University, Chongqing 400016, China; 13598871754@163.com (X.Q.); 13855871847@163.com (Y.L.); 2 Department of Radiology, The First Affiliated Hospital of Chongqing Medical University, Chongqing 400016, China

**Keywords:** magnetic resonance imaging, coil, high-resolution, experimental mice

## Abstract

The clinical magnetic resonance scanner (field strength ≤ 3.0 T) has limited efficacy in the high-resolution imaging of experimental mice. This study introduces a novel magnetic resonance micro-coil designed to enhance the signal-to-noise ratio (SNR) and contrast-to-noise ratio (CNR), thereby improving high-resolution imaging in experimental mice using clinical magnetic resonance scanners. Initially, a phantom was utilized to determine the maximum spatial resolution achievable by the novel micro-coil. Subsequently, 12 C57BL/6JGpt mice were included in this study, and the novel micro-coil was employed for their scanning. A clinical flexible coil was selected for comparative analysis. The scanning methodologies for both coils were consistent. The imaging clarity, noise, and artifacts produced by the two coils on mouse tissues and organs were subjectively evaluated, while the SNR and CNR of the brain, spinal cord, and liver were objectively measured. Differences in the images produced by the two coils were compared. The results indicated that the maximum spatial resolution of the novel micro-coil was 0.2 mm. Furthermore, the subjective evaluation of the images obtained using the novel micro-coil was superior to that of the flexible coil (*p* < 0.05). The SNR and CNR measurements for the brain, spinal cord, and liver using the novel micro-coil were significantly higher than those obtained with the flexible coil (*p* < 0.001). Our study suggests that the novel micro-coil is highly effective in enhancing the image quality of clinical magnetic resonance scanners in experimental mice.

## 1. Introduction

In the continuous development and innovation of medicine, animal research plays an indispensable role [1]. Specifically, studies on life sciences, disease mechanisms, and drug efficacy as well as side effects require animal models for preclinical experiments [2,3,4,5]. Animal magnetic resonance imaging (MRI) is a non-invasive in vivo imaging technology with a high spatial and temporal resolution, enabling real-time and repetitive imaging of rodents such as mice in medical experiments, thereby allowing us to obtain a detailed observation of disease changes and trends in animal models [6,7]. MRI in animals has become an attractive method for evaluating the effects of animal models, and it is widely accepted by medical researchers [8,9]. However, animal MRI faces several challenges. Specialized or ultra-high-field magnetic resonance scanners for animal research are resource-intensive and expensive, limiting their widespread use by medical research institutions [10,11]. Consequently, some magnetic resonance scanners with a field strength of 3.0 T and below have been used for conducting MRI of mouse models with flexible universal coils in clinical settings, but their imaging quality is often suboptimal [12,13].

To improve the high-resolution imaging quality of experimental mice in clinical magnetic resonance scanners, our hospital introduced a 16-channel novel micro-coil to enhance imaging effects and explore its application value in in vivo imaging. It is anticipated that this will establish a specialized animal imaging platform based on clinical magnetic resonance scanners, potentially greatly promoting translational research in clinical environments.

## 2. Materials and Methods

### 2.1. Materials

**Figure 1:**
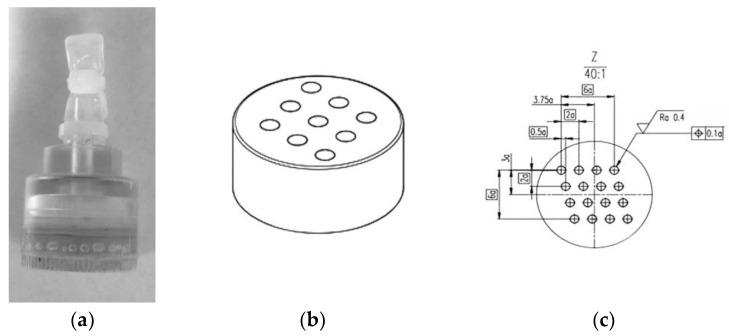
Images of the phantom: appearance and internal structures. (**a**) Appearance of the phantom. (**b**) Stereogram of the 0.5 mm detection structure, 3 × 3 hole array. (**c**) Plan of the 0.2 mm detection structure, 4 × 4 hole array. The diameter and spacing of each hole marked in the figure are 0.2 mm.

We used a phantom with a high-resolution detection structure to determine the maximum spatial resolution of the coils. The phantom was manufactured by Chongqing HAIFU Medical Technology Co., Ltd. in Chongqing, China, and referred to the phantom standard of the American College of Radiology (ACR). The diameter of the cylinder at the bottom of the phantom is 2.2 cm, the column height is 1.8 cm, and the total height of the phantom is 4 cm. The interior of the phantom consists of three groups of hole arrays with circular cross sections that generate signals. The diameters and intervals of the circles in the three groups of hole arrays are 0.8 mm, 0.5 mm, and 0.2 mm, respectively. Its principle involves alternating areas where signals are generated and areas where signals are not, with each area following the same arrangement. This imaging arrangement determines the minimum distinguishable interval. See Figure 1 for details.

**Figure 2:**
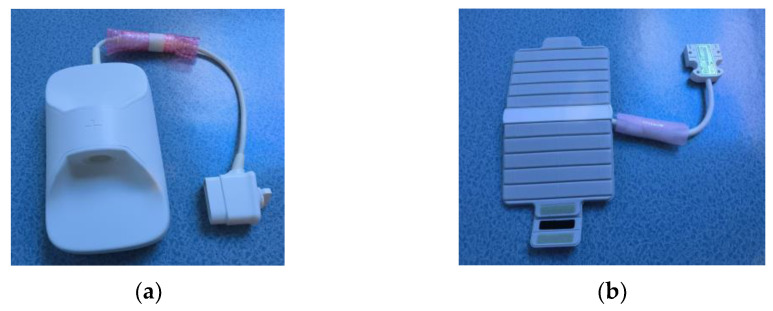
Images of two types of coils. (**a**) The novel micro-coil. (**b**) The flexible coil.

The novel micro-coil (Shanghai Chenguang Medical Technology Co., Ltd., Shanghai, China) is a high-density array coil measuring 40 cm × 20 cm × 14 cm. It features a 16-channel phased array structure. It primarily detects alternating magnetic field signals from mouse tissues, excited by the MR system through the LC resonant circuit, and transmits these signals to the MR system via the transmission line. The coil includes a cylindrical hole (4 cm in diameter and 10 cm in length) that mimics the shape of mice, ensuring a good fit and adequate volume. Figure 2a illustrates the novel micro-coil.

The 16-channel flexible coil (16-s Array, 3.0 T Receive Only, GE Healthcare, Waukesha, WI, USA) is a phased array coil with a wrapping capability, commonly used in clinical settings. Its dimensions are 44 cm × 23 cm × 4 cm, with a wrap diameter ranging from 9.0 cm to 12.5 cm. In the absence of specialized coils, some researchers use the flexible coil to wrap mice for imaging in scientific experiments due to its flexibility. Therefore, in this study, the flexible coil was used as a control to compare the imaging effectiveness of the novel micro-coil in experimental mice. Figure 2b shows the flexible coil.

Additionally, twelve healthy C57BL/6JGpt mice (six males and six females), aged approximately 5 weeks and weighing 22 ± 3 g, were purchased from Chengdu Yaokang Biotechnology Co., Ltd. in Chengdu, China. (license number SCXK (Sichuan) 2020-034). Tribromoethanol (Beijing Yoshida Biotechnology Co., Ltd., Beijing, China) and 1 mL syringes were used. This study was approved by the Ethics Committee of the First Affiliated Hospital of Chongqing.

### 2.2. Magnetic Resonance Scanning Method and Parameter Settings

The examinations were conducted using a GE SIGNA Premier 3.0 T MRI scanner (GE Healthcare, Waukesha, WI, USA). The phantom was positioned at the center of the coils, which were placed at the center of the magnet. Mice were anesthetized with an intraperitoneal injection of 1.25% tribromoethanol (350 mg/kg) prior to examination. The anesthetized mice were placed in the prone position at the center of both the novel micro-coil and the flexible coil. The coils were operating at 127 MHz. The 2D-FSE T2WI sequence was used to scan the transverse and coronal planes of the mouse brain as well as the sagittal planes of the mouse body for two-dimensional imaging, while the 3D-CUBE sequence was used to scan the sagittal plane of the mouse body for three-dimensional imaging. The main parameters for each magnetic resonance scanning sequence are listed in Table 1.

The spatial resolution of each sequence was maintained at the sub-millimeter level. The spatial resolution of the 2D-FSE T2WI sequence was 0.195 mm × 0.195 mm × 0.900 mm, and the spatial resolution of the 3D-CUBE sequence was 0.417 mm × 0.417 mm × 0.400 mm, which is considered isotropic scanning. Following scanning, the images were evaluated both subjectively and objectively using GE’s AW VolumeShare 7 post-processing workstation. The image data obtained from the 3D-CUBE sequence were processed into volumetric rendering (VR) images.

### 2.3. Subjective Evaluation of Images

In this study, two radiologists with over 5 years of experience subjectively evaluated the image quality of the transverse and coronal brain positions and the sagittal body position in terms of noise, artifacts, and clarity, as well as the three-dimensional VR images, using a double-blind method. The evaluation used the 5-point Likert scale [14,15]. The specific evaluation criteria were as follows: One point: very poor image quality with serious artifacts, severe noise, image distortion, or poor signal intensity; tissues and organs are particularly unclear. Two points: poor image quality with obvious artifacts, moderate blurring or low signal intensity, large noise; tissues and organs are not clearly displayed. Three points: medium image quality with slight artifacts or blurring, some noise; tissues and organs are somewhat clearly displayed. Four points: better image quality with fewer artifacts and less noise; tissues and organs are more clearly displayed. Five points: excellent image quality with almost no artifacts and low noise; tissues and organs are particularly clearly displayed. The ratings from the two radiologists for each mouse were averaged to determine the final score for each mouse.

### 2.4. Objective Evaluation of the Images

The SNR and CNR values were determined. The images were processed on the post-processing workstation. Regions of interest (ROIs), approximately 1 mm^2^, were placed on the brain, spinal cord, and liver, and the signal intensity (SI) value and the background noise (SD) were measured. SI represents the average signal intensity of the pixels in the ROI, and SD represents the standard deviation of the pixel signal intensity in the ROI. The copy-and-paste method was employed to ensure consistency in the area of each ROI. The left and right sides of the transverse and coronal brain position were measured and symmetrically averaged, and the signal intensity of the surrounding background was used as a control when calculating the CNR of all tissues. Background signal intensity (SI background) was the average of the signal intensity of four ROIs in the surrounding background area. Background noise (SD noise) was the average of the standard deviation of the signal intensity of four ROIs in the surrounding background area. All ROIs were set on the uniform signal of tissue or surrounding background. The calculation formulas were as follows [16,17]: SNR tissue = SI tissue/SD noise; CNR tissue = (SI tissue − SI background)/SD noise. SI tissue represented the average signal of the tissues, including brain, spinal cord, and liver.

### 2.5. Statistical Methods

All data were statistically analyzed using SPSS 27.0 (IBM, Chicago, IL, USA). In the subjective evaluation, the results from the two radiologists were expressed as mean ± standard deviation, and the Wilcoxon rank-sum test was used. In the objective evaluation, if the measurement data fitted the normal distribution, they were expressed as mean ± standard deviation, and the paired-samples *T*-test was used; if they did not fit the normal distribution, the Wilcoxon rank-sum test was used. The Kappa consistency test was performed for the subjective evaluation results of the two radiologists, with specific criteria as follows: Kappa ≤ 0.4: poor consistency; 0.4 < Kappa ≤ 0.75: good consistency; 0.75 < Kappa ≤ 0.8: very good consistency; Kappa > 0.8: excellent consistency. *p* < 0.05 was considered statistically significant.

## 3. Results

### 3.1. Maximum Spatial Resolution

**Figure 3:**
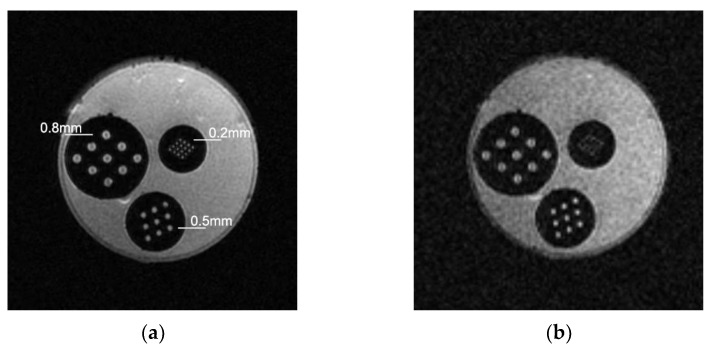
MR images of the resolution phantom recorded using the (**a**) micro-coil and (**b**) flexible coil.

The phantom contained three resolution detection structures: 0.8 mm, 0.5 mm, and 0.2 mm. The novel micro-coil could detect a maximum spatial resolution of 0.2 mm in the phantom. However, the 0.2 mm array was unclear with the flexible coil. As shown in Figure 3.

### 3.2. Subjective Ratings of Two Types of Coils’ Scanning Images

After the scan, the scores from the two radiologists were blinded. The results showed that the subjective scores for the micro-coil on 2D-FSE T2WI images of the transverse and coronal brain positions as well as the sagittal body position of mice were higher than those for the flexible coil. Additionally, the subjective scores for the micro-coil on 3D-CUBE images and VR three-dimensional images of the sagittal body position of mice were higher than those for the flexible coil, with statistical significance (*p* < 0.05). See Table 2 for details.

**Figure 4:**
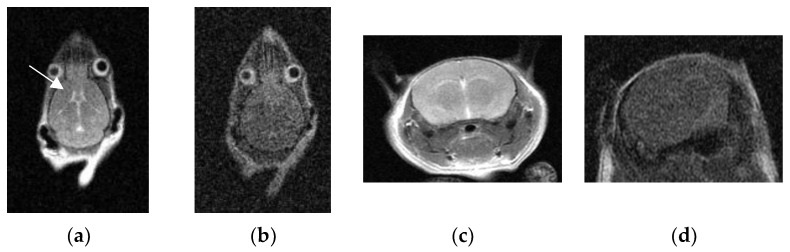
MR images of the mice brain in the 2D-FSE T2WI sequence. (**a**,**c**) Using the micro-coil: low noise, good image contrast, and clear display of cerebrospinal fluid (arrow). (**b**,**d**) Using the flexible coil: high noise, poor image contrast, and almost unable to show cerebrospinal fluid.

**Figure 5:**
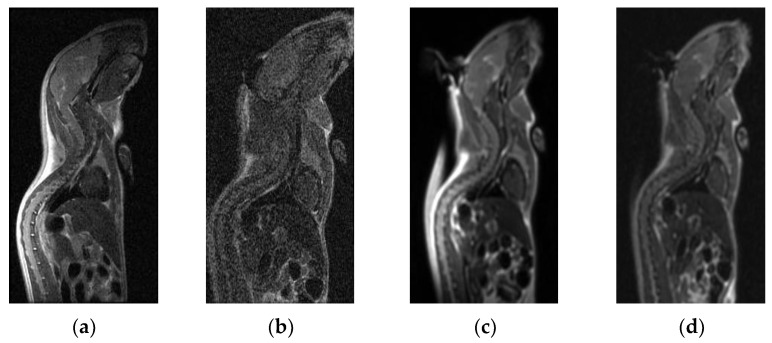
MR images of the sagittal body of mice. (**a**,**c**) Using the novel micro-coil: the image contrast was good, and all tissues and organs were shown more clearly. (**b**,**d**) Using the flexible coil: the image contrast was poor, and the display of various tissues and organs was not as good as that of the micro-coil.

The Kappa values of the two doctors’ subjective scores were both ≥ 0.8 (*p* < 0.05), indicating excellent consistency. See Figure 4 and Figure 5 for the comparison of the two types of coils’ scanning images.

### 3.3. SNR and CNR Values of Two Types of Coils’ Scanning Images

After scanning, the SNR and CNR values for the brain, spinal cord, and liver of the mice were calculated. The results showed that the SNR and CNR of 2D-FSE T2WI images of the transverse and coronal positions of the brain as well as the sagittal position of the body (spinal cord and liver) with the micro-coil were significantly higher than those with the flexible coil (*p* < 0.001). Additionally, the SNR and CNR of 3D-CUBE images of the sagittal position (spinal cord and liver) with the micro-coil were also significantly higher than those with the flexible coil (*p* < 0.001). See Table 3 for details.

### 3.4. VR Three-Dimensional Images

**Figure 6:**
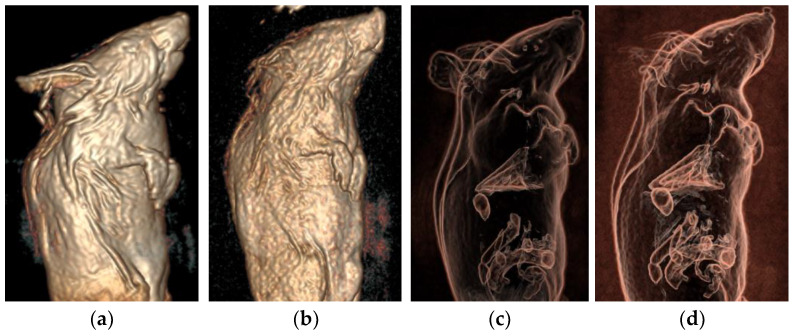
Three-dimensional stereoscopic image of mice VR. (**a**,**c**) Using the novel micro-coil. (**b**,**d**) Using the flexible coil. The quality of the three-dimensional image produced via micro-coil scanning is better than that of the flexible coil.

The 3D stereoscopic images of the mice were created from the 3D-CUBE sequence images, as shown in Figure 6.

## 4. Discussion

The novel micro-coil used in this study clearly distinguishes mouse tissues and organs in both two-dimensional and three-dimensional MR images, exhibiting a high signal-to-noise ratio, high contrast-to-noise ratio, and high spatial resolution. Compared to the flexible coil, the image quality was significantly improved with the novel micro-coil.

In this study, the filling factor was defined as the ratio of mouse volume to coil volume. Consequently, the flexible coil, which had a larger volume, exhibited a smaller filling factor, while the novel micro-coil, possessing a smaller volume, demonstrated a larger filling factor. The larger the filling factor, the greater the SNR. Based on volume estimations of the two coils, the SNR of the novel micro-coil was approximately 11 to 23 times that of the flexible coil. Furthermore, in the 2D-FSE T2WI sequence of our experiment, the SNR for each tissue type (transected brain, coronary brain, spinal cord, and liver) of the novel micro-coil was approximately 2.6, 2.7, 5.8, and 1.4 times that of the flexible coil, respectively. In the 3D-CUBE sequence, the SNR for each tissue type (spinal cord and liver) of the novel micro-coil was approximately 5.4 and 2.2 times that of the flexible coil, respectively. Compared with the previously estimated values, the experimental results were lower. Consequently, we hypothesized that the SNR was additionally influenced by both the tissue type and the magnetic resonance sequence utilized.

Both the micro-coil and flexible coil used in this study have 16 channels, ensuring that they do not exhibit an influence on image quality due to different channel numbers. In clinical settings, flexible coils are typically used as general coils for areas lacking specialized coils or for special requirements due to their flexible operation and broad range of applications [18,19]. Consequently, many researchers choose flexible coils for scientific research [20,21]. Due to the small size of mice, flexible coils cannot couple well with them in MR imaging, resulting in a large gap that leads to poor imaging quality [22]. However, the novel micro-coil can structurally couple well with mice, minimizing surrounding gaps and increasing the filling factor [23], thus enabling better imaging of small mice. Additionally, for small animals (such as mice), in vivo imaging in a clinical 3.0 T magnetic resonance imaging scanner is technically challenging. The smaller the volume, the weaker the MR signal, and the smaller the voxel unit in high-resolution imaging, ultimately resulting in a lower signal-to-noise ratio [24]. The combination of a customized micro-coil and targeted changes to sequence parameters can address this issue, producing high-quality magnetic resonance images.

When using the novel micro-coil for two-dimensional imaging, the field of view (FOV) was 50 mm × 50 mm, the matrix was 256 × 256, and the slice thickness was 0.9 mm, resulting in a spatial resolution of 0.195 mm × 0.195 mm × 0.900 mm and a planar resolution of approximately 0.2 mm, providing more image information [25]. To further improve the spatial resolution of two-dimensional imaging, in a follow-up study, we adjusted the matrix to 384 × 384 while keeping the FOV unchanged. At this point, the spatial resolution was 0.130 mm × 0.130 mm × 0.900 mm. We found that the signal-to-noise ratio of the image decreased sharply. Therefore, we adjusted the number of excitations from 4 to 8 to improve the signal-to-noise ratio and found that the overall image quality improved, but the imaging time was prolonged accordingly. With the increase in imaging time, better anesthesia conditions and higher-level operator scanning techniques are needed to suppress motion artifacts. Additionally, some researchers have performed two-dimensional magnetic resonance imaging of the brain and abdomen of mice, achieving certain imaging effects. However, the spatial resolution was not as ideal as in this study, and there were no studies on sagittal and three-dimensional imaging of the mouse body [26,27].

In this study, the 3D CUBE sequence was used for three-dimensional imaging of mice, achieving isotropic scanning with a high spatial resolution and a high signal-to-noise ratio [28,29]. The spatial resolution was approximately 400 μm, involving volume scanning without slice spacing [30]. This enables the acquisition of reconstructed images of mice in any plane and three-dimensional images, allowing the three-dimensional sequence to be used for measuring target volumes, such as quantifying tumor size [31], thereby facilitating better observation conditions for researchers. Additionally, the 3D CUBE sequence utilizes variable flip angle regrouping RF pulses and zero-pitch scanning, which suppress image blurring, effectively avoid magnetic sensitivity artifacts and partial volume artifacts, and greatly improve the signal-to-noise ratio of the images [32].

This study had certain limitations: (1) Tumor-bearing mice were not included; only healthy mice were selected for imaging. Our future research will aim to strengthen the collaboration with clinical researchers using mouse models and will include more tumor-bearing samples. (2) The mice were not scanned using DWI, PWI, and SWI. Our future studies will further investigate functional MR imaging to provide additional imaging methods for scientific research. (3) This study involved high-resolution imaging of mice using a clinical 3.0 T magnetic resonance scanner. However, the resolution was not comparable to that of ultra-high-field magnetic resonance imaging (tens of micrometers).

## 5. Conclusions

In summary, the use of a novel micro-coil optimizes the imaging effect on experimental mice with a 3.0 T magnetic resonance scanner, enabling a high spatial resolution to be generated in the sub-millimeter range (at least 195 μm). Additionally, the images exhibit a high signal-to-noise ratio and contrast-to-noise ratio, clearly imaging multiple organs of mice and enabling both two-dimensional and three-dimensional imaging. This advancement provides researchers with more options in experiments with animal models, contributing to the development of an effective platform for imaging animals using clinical MR scanners and promoting translational research in clinical settings.

## Figures and Tables

**Table 1:** Parameter values.

Parameter Category	2D-FSE T2WI	3D-CUBE
Repeat Time (ms)	2500	2500
Echo Time (ms)	80	90
Slice Thickness (mm)	0.9	0.4
Matrix	256 × 256	192 × 192
Field of View (mm)	50 × 50	80 × 80
Number of Excitation	4	1
Scanning Time (min:s)	3:43	5:06

**Table 2:** Subjective ratings of two types of coils’ scanning images.

	Novel Micro-Coil	Flexible Coil	Z Value	*p* Value
2D-FSE T2WI tra brain	4.29 ± 0.45	2.63 ± 0.48	−3.095	0.002
2D-FSE T2WI cor brain	4.29 ± 0.45	2.63 ± 0.48	−3.095	0.002
2D-FSE T2WI sag body	4.75 ± 0.45	2.92 ± 0.29	−3.169	0.002
The 3D-CUBE sag body	4.46 ± 0.50	3.17 ± 0.39	−2.859	0.004
VR 3D images body	4.17 ± 0.39	3.08 ± 0.29	−3.127	0.002

**Table 3:** SNR and CNR comparison of scanning images with two types of coils.

Sequence and Location		Novel Micro-Coil	Flexible Coil	T Value	*p* Value
SNR	2D-FSE T2WI tra brain	18.92 ± 1.14	7.24 ± 0.57	27.868	<0.001
	2D-FSE T2WI cor brain	21.52 ± 1.59	7.90 ± 0.83	24.448	<0.001
	2D-FSE T2WI Sag spinal cord	35.87 ± 2.35	6.23 ± 0.57	42.564	<0.001
	2D-FSE T2WI Sag liver	8.61 ± 0.60	6.09 ± 0.65	9.604	<0.001
	3D-CUBE Sag spinal cord	272.55 ± 55.86	50.75 ± 6.37	14.117	<0.001
	3D-CUBE Sag liver	49.92 ± 9.89	22.92 ± 2.69	9.548	<0.001
CNR	2D-FSE T2WI tra brain	16.61 ± 1.08	4.92 ± 0.45	31.355	<0.001
	2D-FSE T2WI cor brain	19.51 ± 1.48	5.62 ± 0.63	28.814	<0.001
	2D-FSE T2WI Sag spinal cord	33.86 ± 2.23	3.96 ± 0.37	47.281	<0.001
	2D-FSE T2WI Sag liver	6.60 ± 0.50	3.82 ± 0.45	14.525	<0.001
	3D-CUBE Sag spinal cord	270.55 ± 55.67	48.71 ± 6.03	14.143	<0.001
	3D-CUBE Sag liver	47.92 ± 9.68	20.88 ± 2.39	9.769	<0.001

## Data Availability

The raw data supporting the findings of this study are available from the corresponding authors upon reasonable request.
